# Extracellular Vesicles Derived From Stem Cells in Intervertebral Disc Degeneration

**DOI:** 10.3389/fcell.2021.793363

**Published:** 2022-01-13

**Authors:** Xinjie Wu, Wei Sun

**Affiliations:** ^1^ Peking University China-Japan Friendship School of Clinical Medicine, Beijing, China; ^2^ Department of Orthopedic Surgery, China-Japan Friendship Hospital, Beijing, China

**Keywords:** nucleus pulposus cells, annulus fibrosus, cartilage endplate, intervertebral disc degeneration, extracellular vesicles

## Abstract

Intervertebral disc degeneration (IVDD) is the leading cause of low back pain related to degradation of cartilaginous tissues, mainly resulting from oxidative stress, cell apoptosis, and extracellular matrix degradation. Extracellular vesicles (EVs) exist in all bodily fluids and can be produced by all types of cells. Stem cell-derived EVs (SC-EVs), which are the main paracrine components of stem cells, have gained significant attention in the field of regenerative medicine. Over the past years, accumulating evidence indicates the therapeutic and diagnostic potentials of EVs in IVDD. The main mechanisms involve the induction of regenerative phenotypes, apoptosis alleviation, and immune modulation. In addition, the efficiency of SC-EVs can be enhanced by choosing appropriate donor cells and cell phenotypes, optimizing cell culture conditions, or engineering EVs to deliver drugs and targeting molecules. Given the importance and novelty of SC-EVs, we give an overview of SC-EVs and discuss the roles of SC-EVs in IVDD.

## Introduction

Intervertebral disc degeneration (IVDD) is an age-related disease associated with various factors. The pathology involves the decrease of nucleus pulposus cells (NPCs) and extracellular matrix (ECM), aging of the annulus fibrosus (AF), and calcification of cartilage endplates (CEPs) (Feng et al., 2016; Vo et al., 2016). The NPCs maintain homeostasis by producing components of ECM, such as proteoglycan and type II collagen (Zhang et al., 2021a). The highly specialized ECM is essential for stabilizing the biomechanical equilibrium and structure of IVD. Furthermore, mechanical damage and inflammation are involved in IVDD, disturbing ECM metabolism (Ruiz-Fernández et al., 2019; Zhang et al., 2021b). Hence, the dysfunction or loss of such cells could disrupt the ECM formation, which is significantly related to the degradation of cartilaginous tissues.

IVDD is a leading chronic joint disease among the elderly population, seriously affecting the life quality of people (Goh et al., 2019; Tryfonidou et al., 2020). Despite augmented studies focused on investigating the pathogenesis of the disease, many of the underlying mechanisms involved in IVDD remain largely unknown. Compared to terminally differentiated cells, stem cells (SCs) exhibit beneficial characteristics including the capability of self-renewal and the potential to differentiate towards multilineage cells. However, despite the promises and benefits, there are still many challenges facing SC applications, such as their variability, scalability, and delivery, as well as ethical and safety issues (Sakai and Andersson, 2015; Sakai and Schol, 2017; Clouet et al., 2019). Extracellular vesicles (EVs) are particles released from cells that are delimited by a lipid bilayer and cannot replicate (Théry et al., 2018). Recently, SC-derived EVs (SC-EVs), preventing safety concerns associated with cell therapy, have emerged as novel drug and gene delivery tools. SC-EVs have been implicated in good therapeutic effects against several types of musculoskeletal disorders including osteoarthritis, congenital myopathies, IVDD, and so on (Bari et al., 2018; Bier et al., 2018; Murphy et al., 2018; Zhang et al., 2020a). In the present review, we provide an overview of EVs and summarize emerging literature to discuss the roles of SC-EVs in IVDD.

## Overview of Extracellular Vesicles

### Definition and Classification

EVs are an umbrella definition of lipid bilayer-delimited particles that are naturally released or shed from almost all organisms and cell types studied. Also, EVs are not cells. Therefore, they cannot replicate because they are not living. Since EV release was first observed in sheep reticulocytes in 1983, a growing number of studies have shown EVs as a pivotal mechanism of the intercellular communication network (Pan and Johnstone, 1983).

The exact classification of EVs is still evolving. In studies on degenerative joint diseases, the term exosome is commonly used. Exosomes were deemed to have an endocytic origin and their diameters between 30 and 150 nm (Hessvik and Llorente, 2018). However, some vesicles with diameters larger than 150 nm can also be generated by the endosomal pathway (Ronquist and Brody, 1985), and some with diameters smaller than 150 nm can bleb directly from the plasma membrane (Nabhan et al., 2012). Therefore, the classification of EVs based on size must be considered carefully. Due to significant size overlap, similarities in composition, and lack of specific markers, terms for EV subtypes are recommended to refer to physical traits such as size [for instance, respectively, <100 or <200 nm (small EVs), or >200 nm (large and/or medium EVs)], density (low, middle, high), biochemical composition, or descriptions of conditions or cell of origin according to minimal information for studies of extracellular vesicles (MISEV) 2018 (Théry et al., 2018).

### Separation and Enrichment

Isolation of EVs from specific tissues, biological fluids, or cells is the first step carried out in all studies on EVs. However, it is not realistic to achieve absolute purification or complete isolation of EVs from other entities. Therefore, it is recommended to use the terms “separation” and “enrichment”.

Hitherto, there is no consensus on a “gold standard” method for EV separation (Gardiner et al., 2016). The optimal method of separation should be chosen based on the downstream applications and scientific issues. According to a worldwide survey published in 2016, the most common downstream applications were *in vitro* functional analyses, transcriptome analysis, proteomic analysis, *in vivo* functional analyses, and lipidomic analysis (Gardiner et al., 2016). In the survey, researchers coping with complex biological fluids and/or performing proteomic analysis usually tend to utilize more elaborate strategies than those who separate EVs from culture media and/or use flow cytometry for EVs analysis.

Ultracentrifugation (UC) remains the most utilized primary separation technique for EVs, regardless of the starting material used (Gardiner et al., 2016). However, there still exist some problems and drawbacks such as lengthy timescales, the requirement of a large number of cells or biological fluids, and non-vesicular macromolecule contamination (Webber and Clayton, 2013; Gupta et al., 2018). Also, UC can induce aggregation of EVs, which may lead to erroneous interpretation during analysis (Linares et al., 2015). EVs are considered a drug delivery tool and gain considerable interest. However, the efficacy of EVs depends greatly on biodistribution and clearance. Interestingly, different separation methods may result in different results. For instance, some studies revealed that UC-separated EVs have a higher rate of accumulation, affecting the biodistribution and delivery efficiency (Nordin et al., 2015; Smyth et al., 2015).

To overcome the limitations of conventional techniques, recently emerging EV separation techniques have been developed including size-, charge-, and affinity-based techniques. For instance, size-exclusion chromatography (SEC) applications have become more prominent since 2010 (Liangsupree et al., 2021). SEC has been utilized for the separation of EVs from a large variety of origins from both prokaryotes and eukaryotes, involving cells, plasma and serum, urine, synovial fluid, cerebrospinal fluids, and others (Foers et al., 2018; Guan et al., 2020; Povero et al., 2020). Several studies have also shown the superiority of SEC, allowing purer EV preparations with easy implementation (Allelein et al., 2021; Tsai et al., 2021). For instance, when EVs were separated from plasma *via* the UC technique, high-density lipoproteins (HDLs) were co-separated, because HDLs share a similar density range with EVs. With the SEC technique, this issue could be addressed due to HDLs being smaller than EVs. However, this method is time-consuming and not suitable for large sample processing.

Other methods include ultrafiltration, flow field-flow fractionation, anion-exchange chromatography, electrophoresis, dielectrophoresis, affinity chromatography, immunocapture, and so on (Heath et al., 2018; Stranska et al., 2018; Lewis et al., 2019; Logozzi et al., 2020; Wu et al., 2020; Zhang et al., 2021c). In the included studies, the most commonly used method is ultracentrifugation. Among the included studies, one study reported a pilot production process for a freeze-dried, “ready-off-the-shelf” and free soluble powder containing EVs and proteins *via* ultrafiltration (Bari et al., 2018). This may contribute to transforming MSC-secretome into a pharmaceutical product for its large-scale production. Of note, it is still recommended to use combinations of methods, which may outperform single-method approaches.

### Characterization

After separation, it is extremely important to assess EV characterization *via* multiple, complementary approaches, confirming that biomarkers or functions are related to EVs rather than other co-separated materials. So far, there is no single perfect quantification approach for EVs. The most frequently used are total particle number and total protein amount. Alternatively, quantification of total lipid can also be considered. Nanoparticle tracking analysis (NTA) is developed to assess particle concentration and size distribution. Unlike measuring bulk scattered light from EVs *via* dynamic light scattering (DLS), NTA measures individual particle scattering (Shao et al., 2018). For accurate quantification, however, it requires accurate optimization of camera and analysis settings. Of note, large EVs (>400 nm) and very small EVs (<50 nm) are not well quantified by all NTA. Tunable resistive pulse sensing can be a substitute to NTA for a wide range of sizes, depending on pore diameter (Vogel et al., 2016; Maas et al., 2017). According to the size of EVs, standard or high-resolution flow cytometry can also be applied (Nolan and Duggan, 2018; Tertel et al., 2020).

**FIGURE 1:**
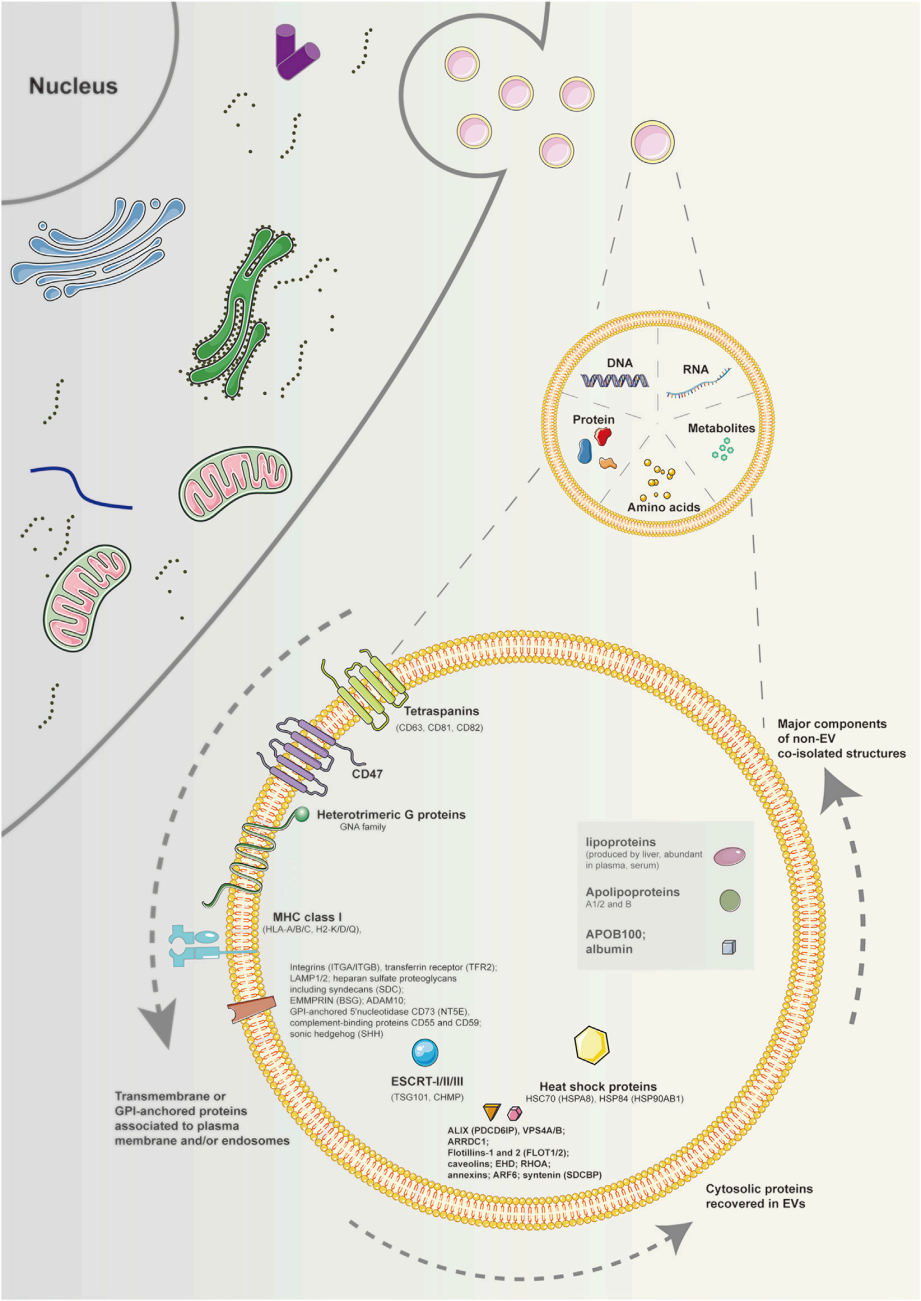
Schematic diagram of components and markers of extracellular vesicles. EVs are particles naturally released from the cell that are delimited by a lipid bilayer and cannot replicate. The components of EVs contain DNAs, RNAs, proteins, amino acids, metabolites, and so on. Regarding protein markers of EVs, at least one protein of transmembrane or GPI-anchored proteins associated to plasma membrane and/or endosomes, cytosolic proteins recovered in EVs, and major components of non-EV co-isolated structures must be analyzed to demonstrate the EV nature and the degree of purity of EV preparation. Note: EVs, extracellular vesicles; GPI, glycosylphosphatidylinositol.

MISEV 2018 highlights three categories of markers (transmembrane or GPI-anchored proteins associated with the plasma membrane and/or endosomes; cytosolic proteins recovered in EVs; major components of non-EV co-isolated structures), which investigated necessarily in all bulk EV preparations to show the presence of EVs and evaluate their purity from common contaminants (Théry et al., 2018) (Figure 1). However, there are no suggestions for universal “negative controls” related to a particular subtype of EVs. Western blotting is the most common method to evaluate the presence of proteins in EV preparations. However, it is easier applied for cells than biofluids. Alternatively, flow cytometry of EVs decorating beads of bulk EV populations can be utilized, but with appropriate negative controls including antibodies alone or isotype controls used with caution (Pospichalova et al., 2015). In addition, mass spectrometry is also an economical and accessible approach, enabling the assessment of many proteins at once (Bandu et al., 2019).

Techniques allowing visualization of single vesicles at high resolution are recommended to verify the characterization of EVs. Transmission electron microscopy (TEM) is a popular technique for characterizing EVs. It could provide superior resolution, with capabilities to image less than 1 nm objects (Mohammadian et al., 2020). Moreover, contrasting and embedding in heavy metal stains such as osmium tetroxide and uranyl acetate can be used to maintain the morphology of the lipid membrane. Also, TEM can be combined with immunogold labeling (immuno-EM) to visualize molecular characterization (Street et al., 2012). Other similar methods include scanning electron microscopy, cryo-electron microscopy, and atomic force microscopy (Shao et al., 2018).

Importantly, an increasing number of studies found that the topology of EV components can fundamentally influence the biological functions of EVs (Cvjetkovic et al., 2016; De la Torre-Escudero et al., 2019). Some active components of EVs are localized on the EV surface and sensitive to digestion. Hence, it is recommended to perform mild digestions, permeabilizations, or antibody studies to determine the actual topology of active components. Alternatively, flow cytometry and fluorescence microscopy with antibodies targeting EV membranal or cytoplasmic epitopes. In addition, as mentioned above, TEM with immuno-EM can also be applied to aid in determining differences in topology among different EVs. In the included studies, the most commonly used methods are TEM and NTA combined with western blotting.

### Components and Functions

Depending on the cell of origin, EVs can contain various components of cells, including DNA, RNA, lipids, metabolites, and proteins (Kalluri and LeBleu, 2020). The cargo carried by EVs may mirror the parental cell physiological or pathological conditions, involving different diseases as well as stages of a disease. Such intrinsic properties of EVs have facilitated their potential utility in therapy and diagnosis for many diseases, including degenerative joint diseases. Also, EVs can be engineered to deliver many effective constituents to target cells, including short interfering RNAs, antisense oligonucleotides, chemotherapeutic agents, and immune modulators. (Figure 1).

Since the first public online database of EVs, Exocarta (http://www.exocarta.org), is accessible, a growing number of databases focused on EVs have been established (Keerthikumar et al., 2016). For instance, ESBL (https://hpcwebapps.cit.nih.gov/ESBL/Database/Exosome) is a database of urinary EVs proteins based on published protein mass spectrometry data (Pisitkun et al., 2004). Vesiclepedia (http://www.microvesicles.org) is another database of RNA, proteins, lipids, and metabolites identified in EVs from both published and unpublished studies (Pathan et al., 2019). Now, Vesiclepedia holds data obtained from 1254 EV studies, 38,146 RNA entries, 349,988 protein entries, and 639 lipid/metabolite entries. Recently, ISEV established an updatable tool, the EV-TRACK knowledgebase, to encourage researchers to report details of EVs studies, ensuring the transparency and reproducibility of procedures (EV-TRACK ConsortiumVan Deun et al., 2017). In this way, it may promote researchers to put MISEV 2018 guidelines into practice.

## Stem Cells and Stem Cell-Derived Extracellular Vesicles

With characteristics of self-renewal and differentiation potentials, SCs have been applied successfully for treating a wide range of musculoskeletal disorders in preclinical and clinical studies (Alcaraz et al., 2019). However, some issues for direct SC applications still exist. For instance, cell survival is unpredictable after cell injection, which is of importance to sustain the effects due to intercellular interactions. It has been reported that mesenchymal SCs (MSCs) inoculated into the damage site demonstrated low survival incidence and loss of function after only 1 week (Rani et al., 2015). Also, the longevity issue of SCs may be related to the status of donors and the microenvironment for SC survival, resulting in unstable effects in treatment. Other possible encountered issues include storage, immunoreaction, occlusion, gene mutation, and tumorigenesis or tumor promotion (Kim et al., 2021).

SC-EVs, as the main paracrine mediators, overcome most limitations of SCs. SC-EVs, an approach of cell-free therapy, different from therapy based on whole cells, are much easier to manage and safer because of lower quantities of membrane-bound proteins such as MHC molecules and their inability of tumorigenesis directly (Keshtkar et al., 2018). Previous studies have shown SC-EVs to traffic SC associated transcription factors including Nanog, Oct-4, HoxB4, and Rex-1 operating at the level of pluripotent SCs (Ratajczak et al., 2006). Furthermore, direct effectors of SC phenotype such as Wnt, *ß*-catenin, and Hedgehog have also been identified on SC-EVs besides several other components (Nawaz et al., 2016). These are considered to be potential factors in SCs biology. As a result, SC-EVs have been used for the treatment of various diseases in preclinical and clinical studies *via* induction of regenerative phenotypes, apoptosis alleviation, and immune modulation (Yin et al., 2020). In addition, the efficiency of SC-EVs can be enhanced by choosing appropriate donor cells and cell phenotypes, optimizing cell culture conditions, or engineering EVs to deliver drugs and targeting molecules.

## The Roles of Stem Cell-Derived Extracellular Vesicles in Intervertebral Disc Degeneration

**FIGURE 2:**
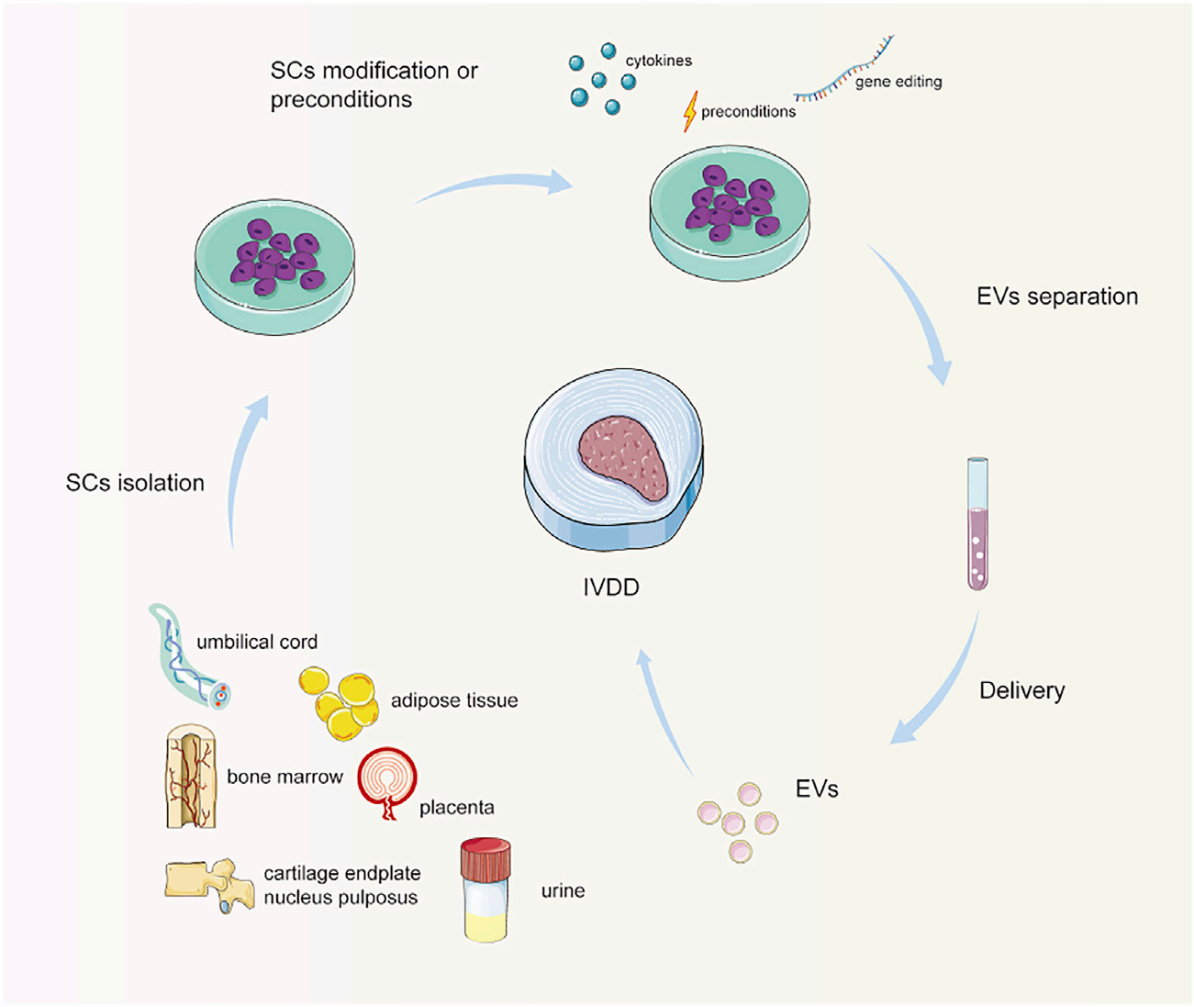
Schematic diagram of stem cell-derived extracellular vesicle separation and delivery. Stem cells are isolated and cultured from different origins. Optionally, stem cells are preconditioned or modified, especially genetically engineered. Then, EVs are separated and enriched, and delivered to the IVD for treatment. Note: SCs, stem cells; EVs, extracellular vesicles; IVD, intervertebral disc.

MscMSC is one of the main cell sources of tissue regeneration because of its potential for multidirectional differentiation and self-renewal. MSCs can produce various kinds of EVs, potentially restoring an extensive range of damaged or diseased tissues and organs, such as lungs, blood vessels, esophagus bowel, as well as bone reconstruction after injury (Tsiapalis and O'Driscoll, 2020). For instance, it was reported that miR-155–5 p in MSC-EVs could enhance proliferation and migration, attenuate apoptosis, and modulate ECM secretion in chondrocytes (Wang et al., 2021). *In vivo*, MSC-EVs can effectively attenuate osteoarthritis progression and protect cartilage from degeneration (Woo et al., 2020). Herein, the most investigated type of MSCs to separate EVs to treat IVDD is bone marrow-derived MSCs (BM-MSCs) due to their easy accessibility. Other sources include the umbilical cord, adipose tissue, placenta, urine, cartilage endplate, and nucleus pulposus (Figure 2).

### Apoptosis, Senescence, and Proliferation

Excessive apoptosis and senescence of NPCs have been proven to be involved in the process of IVDD, considered as the potential therapeutic target (Chen et al., 2016). Recently, SC-EVs have been applied to deliver EV cargos to suppress apoptosis and senescence as well as promote proliferation both *in vitro* and *in vivo* (Lu et al., 2017; Cheng et al., 2018). It was reported that the beneficial effects of SC-EVs were mainly mediated by phosphoinositide 3-kinase (PI3K)-protein kinase B (Akt) pathway, mitogen-activated protein kinase (MAPK)-extracellular signal-regulated kinase (ERK) pathway, Wnt pathway, and transforming growth factor-beta (TGF-β) pathway (Cheng et al., 2018; Liao et al., 2019; Qi et al., 2019; Li et al., 2020a; Zhu et al., 2020a; Xiang et al., 2020; Luo et al., 2021a; Guo et al., 2021). For instance, SC-EVs resisted senescence and promoted NPC proliferation by delivery of matrilin-3 (MATN3) activating TGF-*β* (Guo et al., 2021). After intradiscal injection of SC-EVs in an animal IVDD model, NPCs apoptosis, senescence, and IVDD were significantly alleviated (Cheng et al., 2018; Sun et al., 2021). In addition, using a 3D *in vitro* model, Hingert et al. explored the effects of SC-EVs on degenerated disc cells (DCs), and the results demonstrated an over 50% increase in cell proliferation and decrease in cellular apoptosis (Hingert et al., 2020).

Previous studies have shown the important role of lactic acid and abnormal PH levels in IVD, leading to decreased permeability of CEPs (Malandrino et al., 2014; Xiao et al., 2020). Compared with NPCs cultured at pH 7.1–7.3, proliferation activity of counterparts cultured at pH 5.9–6.7 decreased significantly (Li et al., 2020b). However, SC-EVs reversed the adverse effects of acidic PH on proliferation and apoptosis (Li et al., 2020b). Until now, it is not clear whether the acidic microenvironment is a result or a cause of the degeneration. Hence, further studies need to investigate the relationship between PH and IVDD as well as the mechanism of beneficial effects of SC-EVs.

Interestingly, as the source of EVs, cartilage endplate-derived SCs (CESCs) could not only inhibit apoptosis *via* activation of the PI3K/AKT/autophagy signaling pathway but also promote the invasion, migration, and differentiation of themselves *via* the hypoxia-inducible factor 1-alpha (HIF-1α)/Wnt pathway, increasing expression of GATA binding protein 4 (GATA4) and TGF-*β* (Luo et al., 2021a; Luo et al., 2021b). In addition, the apoptosis of NPCs and IVDD was more attenuated when stimulated by normal CESC-derived EVs than degenerated ones *in vitro* and *in vivo* (Luo et al., 2021a). Of note, these studies did not conduct any sorting analysis to remove CESCs from general CEs. Additionally, IVD stem and progenitor cells are still debated (Wang et al., 2015). The evidence underlines the critical role of the state of cells (Table 1).

**TABLE 1:** Details of stem cell-derived extracellular vesicles for intervertebral disc degeneration.

References	Sources	EV type	Separation method	EV characterization	EV markers	Target	Study design	Animal model	Cargo of EVs	Dosage of EVs	Results
Bari et al. (Bari et al., 2018) becoming cytotoxic to NPCs at a concentration of over 50 mg/ml	Human ASCs	Small EVs (40–120 nm) and medium/large EVs (250–1,000 nm)	Ultrafiltration	1. SE2. 2 DLS. 3. NTA	NA	NPCs	*In vitro*	NA	NA	5, 12.5, 25, 50, 75, 100, 150, 200 mg/ml	1. counteracting the oxidative stress damage induced by H_2_O_2_ on NPCs at concentrations between 5 and 50 mg/ml
Lu et al. (Lu et al., 2017)	Human BM-MSCs	Small Evs (30–100 nm)	Ultracentrifugation	1. TEM. 2. WB	1. EVs marker: CD63, TSG101. 2. Negative control: Calnexin	NPCs	*In vitro*	NA	NA	50 μg/ml	1. promoting NPCs proliferation. 2. increasing matrix synthesis and protection genes. 3. decreasing degradation-related genes
Cheng et al. (Cheng et al., 2018)	Human BM-MSCs	Small Evs (30–100 nm)	Ultracentrifugation	1. NTA. 2. TEM. 3. WB	1. EVs marker: Alix, TSG101, CD9, CD63. 2. Negative control: NA	NPCs	*In vitro* and *in vivo*	Rat	miR-21	1. *In vitro*: 1 μg/ml. 2. *In vivo*: 1.5 × 10^6^/2 μL	1. *In vitro*: miR-21 in SC-EVs suppressing NPCs apoptosis by targeting PTEN through PI3K- AKT pathway. 2. *In vivo*: Intradiscal injection of MSC-exosomes alleviating NPCs apoptosis and IVDD
Li et al. (Li et al., 2020a)	Human BM-MSCs	Small Evs (100 nm)	Separation reagent kit	1. TEM. 2. FC	1. EVs marker: CD9, CD63. 2. Negative control: NA	AF cells	*In vitro*	NA	NA	NA	1. promoting AF cells proliferation. 2. inhibiting IL-1β-induced inflammation and apoptosis of AF cells by suppressing autophagy *via* activating PI3K/AKT/mTOR signaling pathway
Xiang et al. (Xiang et al., 2020)	Human USCs	Small Evs (50–100 nm)	Ultracentrifugation	1. NTA. 2. TEM. 3. WB	1. EVs marker: CD63, TSG101. 2. Negative control: Calnexin	NPCs	*In vitro* and *in vivo*	Rat	NA	10, 50, 100 μg/ml	1. *In vitro*: improving ER stress responses and inhibiting excessive activation of the UPR as well as cell apoptosis and disc degeneration *via* AKT and ERK signaling pathways. 2. *In vivo*: delaying IVDD by reducing ER-stress associated apoptosis
Luo et al. (Luo et al., 2021a)	Rat CESCs	Small Evs (mean 93 nm)	Ultracentrifugation	1. TEM. 2. NTA. 3. WB	1. EVs marker: CD9, CD63, CD81, Alix, TSG101. 2. Negative control: NA	NPCs	*In vitro* and *in vivo*	Rat	NA	40 μg/ml	1. *In vitro:*inhibiting apoptosis *via* activation of the PI3K/AKT/autophagy signaling pathway. 2. *In vivo*: delaying IVDD progression
Zhu et al. (Zhu et al., 2020a)	Murine BM-MSCs	Small Evs (80 nm)	Ultracentrifugation	1. TEM. 2. NTA. 3. WB	1. EVs marker: CD63, TSG101. 2. Negative control: Calnexin	NPCs	*In vitro*	NA	miR-142–3p	50 μg/ml	1. EVs miR-142–3p alleviating NPCs injury through suppressing MAPK signaling by targeting MLK3
Qi et al. (Qi et al., 2019)	Human UC-MSCs	Small EVs (30–200 nm)	Ultracentrifugation	1. TEM	NA	NP-MSCs	*In vitro*	NA	miRNAs	NA	1. protecting NPMSCs from high glucose-induced ECM degradation *via* the p38 MAPK pathway
Guo et al. (Guo et al., 2021)	Human USCs	Small Evs (49.7 ± 7.3 nm)	Ultracentrifugation	1. NTA. 2. TEM. 3. WB	1. EVs marker: CD63, TSG101. 2. Negative control: Calnexin	NPCs	*In vitro* and *in vivo*	Rat	MATN3	1. *In vitro*: 100 μg/ml. 2. *In vivo*: 2μL, 100 μg/ml	1. *In vitro*: resisting senescence and promoting NPCs proliferation and ECM synthesis by delivery of MATN3. 2. *In vivo*: alleviating IVDD by activating TGF-β
Hingert et al. (Hingert et al., 2020)	Human BM-MSCs	Small Evs (<200 nm)	Ultracentrifugation	1. NTA. 2. FC. 3. TEM. 4. WB	1. EVs marker: CD9, CD81, CD63, flotillin-1. 2. Negative control: Grp94, Tom20	DCs	*In vitro*	NA	NA	5 × 10^10^/ml	1. increasing cell proliferation and decreasing apoptosis. 2. expediting chondrogenesis
Li et al. (Li et al., 2020b)	Human BM-MSCs	Small Evs (125 nm)	Ultracentrifugation	1. NTA. 2. TEM. 3. WB	1. EVs marker: CD63, TSG101. 2. Negative control: NA	NPCs	*In vitro*	NA	NA	1, 5, 10, 15, 20, 25, 30 μg/ml	1. promoting extracellular matrix synthesis and reducing degradation. 2. promoting NPCs proliferation and protecting NPCs from acidic pH-induced apoptosis
Luo et al. (Luo et al., 2021b)	Rat CESCs	Small EVs	Ultracentrifugation	1. TEM. 2. WB	1. EVs marker: CD9, CD63, TSG101. 2. Negative control: NA	CESCs	*In vitro* and *in vivo*	Rat	NA	1. *In vitro*: 40 μg/ml. 2. *In vivo*: 20 μL, 10^5^/ml	1. *In vitro*: promoting the invasion, migration, and differentiation *via* the HIF-1*α*/Wnt pathway, increasing expression of GATA4 and TGF-*β*. 2. *In vivo*: promoting the migration of CESCs into the IVD and transformation into NPCs and inhibiting IVDD
Zhu et al. (Zhu et al., 2020b)	Rat BM-MSCs	Small Evs (109.3 nm)	Ultracentrifugation	1. TEM. 2. NTA. 3. WB	1. EVs marker: CD9, CD63, CD81. 2. Negative control: NA	NPCs	*In vitro*	NA	miR-532–5p	NA	1. suppressing apoptosis, ECM degradation, and fibrosis deposition in NPCs through the delivery of miR-532–5p *via* targeting RASSF5
Cui et al. (Cui and Zhang, 2021)	Human BM-MSCs	Small EVs (30–150 nm)	Ultracentrifugation	1. TEM. 2. NTA. 3. WB	1. EVs marker: CD9, CD63, TSG101. 2. Negative control: Calnexin	NPCs	*In vitro* and *in vivo*	Rat	miR-129–5p	1. *In vitro*: 100 μg/ml. 2. *In vivo*: 2 μL, 100 μg/ml	1. *In vitro*: miR-129–5p in MSC-EVs decreasing apoptosis, ECM degradation, and M1 polarization of macrophages *via* targeting LRG1 and suppressing the p38 MAPK signaling pathway in NPCs. 2. *In vivo*: relieving IDD *via* inhibition of the LRG1/p38 MAPK signaling
Xing et al. (Xing et al., 2021)	Rat ASCs	Small EVs (30–150 nm)	Ultracentrifugation	1. TEM. 2. NTA. 3. WB	1. EVs marker: Alix, TSG101. 2. Negative control: Calnexin	NPCs	*In vitro* and *in vivo*	Rat	NA	1. *In vitro*:1 μg. 2. *In vivo*: 1 μg	1. *In vitro*: regulating matrix synthesis and degradation and inhibiting pyroptosis by mitigating the inflammatory response. 2. *In vivo*: maintaining early IVD microenvironment homeostasis and ameliorating IVDD.
Xia et al. (Xia et al., 2019)	Murine BM-MSCs	Small Evs (50–130 nm)	Ultracentrifugation	1. DLS. 2. TEM. 3. WB	1. EVs marker: CD9, TSG101, CD63 2. Negative control: NA	NPCs	*In vitro* and *in vivo*	Rabbit	NA	1. *In vitro*: 100 μg/ml. 2. *In vivo*: 15 μL, 1 μg/1ul	1. *In vitro*: attenuating apoptosis and mitochondrial dysfunction, dampening inflammatory marker expression and matrix degradation, suppressing NLRP3 inflammasome activation.2. *In vivo*: attenuating IVDD progression
Zhang et al. (Zhang et al., 2020b)	Human MSCs	Small Evs (100 nm)	Ultracentrifugation	1. TEM. 2. DLS. 3. WB	1. EVs marker: CD9, CD63, CD81, TSG101. 2. Negative control: GM130, Calnexin	NPCs	*In vitro* and *in vivo*	Mouse	miR-410	20 μg/ml	1. *In vitro*: SC-EVs derived miR410 inhibiting pyroptosis by targeting NLRP3. 2. *In vivo*: inhibiting pyroptosis in IVDD model
Xie et al. (Xie et al., 2020)	Human BM-MSCs	Small Evs (30- 200 nm)	Ultracentrifugation	1. TEM. 2. DLS. 3. WB	1. EVs marker: CD9, CD63, TSG101. 2. Negative control: NA	EPCs	*In vitro* and *in vivo*	Rat	miR-31-5p	NA	1. *In vitro:* miR-31-5p inhibiting apoptosis and calcification in EPCs under oxidative stress by targeting ATF6. 2. *In vivo*: ameliorating IVDD
Yuan et al. (Yuan et al., 2020)	Human PLMSCs	Small Evs (30- 150 nm)	Ultracentrifugation	1. NTA. 2. TEM. 3. WB	1. EVs marker: CD9, CD63. 2. Negative control: NA	NPCs	*In vitro* and *in vivo*	Mouse	miR-4450 inhibitor	1. *In vitro* 1 × 10^10^/ml. 2. *In vivo*: 2 μl. 1 × 10^10^/ml	1. *In vitro* : miR-4450 inhibitor increasing proliferation and migration while decreasing apoptosis by upregulating ZNF121. 2. *In vivo*: retarding IVDD damage
Sun et al. (Sun et al., 2021)	Human iMSCs	Small EVs (80-200 nm)	Ultracentrifugation	1. TEM. 2. NTA. 3. WB	1. EVs marker: CD9, CD63, TSG101. 2. Negative control: GM130, actin	NPCs	*In vitro* and *in vivo*	Rat	miR-105-5p	1. *In vitro*: 1 × 10^10^/ml. 2. *In vivo:* 2 μl. 1 × 10^10^/ml	1. *In vitro* : downregulating PDE4D expression and activating the Sirt6 signaling pathway by delivering miR-105-5p to senescent NPCs, 2. *In viv*o: ameliorating the progression of IVDD and the senescence
Wen et al. (Wen et al., 2021)	Human BM-MSCs	Small EVs (80 nm)	Ultracentrifugation	1. TEM. 2. NTA. 3. WB	1. EVs marker: CD68, CD81, TSG101. 2. Negative control: Calnexin	NPCs	*In vitro* and *in vivo*	Mouse	miR-199a	1. *In vitro*: 20×10^−6^M. 2. *In vivo*: 100 μg /ml	1. *In vitro:* promoting proliferation and inhibiting apoptosis and senescence *via* miR-199a targeting GREM1 to downregulate the TGF-*β* pathway. 2. *In vivo*: promoting the repair of IVDD
Yuan et al. (Yuan et al., 2021)	Human UC-MSCs	Small EVs (65 ± 15 nm)	Ultracentrifugation	1. TEM. 2. NTA. 3. WB	1. EVs marker: CD9, CD63 TSG101 2. Negative control: NA	NPC	*In vitro*	NA	miR-26a-5p	1 μg /ml	1. miR-26a-5p inhibit the ETTL14/NLRP3 pathway to prevent pyroptosis in NPCs
Liao et al. (Liao et al., 2021a)	Human BM-MSCs	Small EVs (80-200 nm)	Ultracentrifugation	1. TEM. 2. NTA. 3. WB	1. EVs marker: CD63, Alix. 2. Negative control: Calnexin	NPCs	*In vitro* and *in vivo*	Rat	Cavin-2	1. *In vitro:* 100 μg/ml. 2. *ex vivo*: 2 μl, 100 μg/ml	1. *In vitro*: protecting against TNF-α-induced NPCs pyroptosis. 2. retarding the progression of IVDD
Liao et al. (Liao et al., 2021b)	Human BM-MSCs	Small Evs (100 nm)	Ultracentrifugation	1. TEM. 2. NTA. 3. WB	1. EVs marker: CD63, Alix. 2. Negative control: Calnexin	NPCs	*In vitro* and *in vivo*	Rat	ITIH4	1. *In vitro*: 100 μg/ml. 2. *in vivo*: 2 μl, 100 μg/ml	1. *In vitro*: Metformin promotes MSC-derived EVs secretion *via* the autophagy-related pathway improved effects on senescence. 2. *In vivo*: ameliorating IVD cells senescence

ASCs, adipose-derived mesenchymal stromal cells; AF, annulus fibrosus; BM-MSCs, bone marrow-derived mesenchymal stem cells; CESCs, cartilage endplate-derived stem cells; DCs, disc cells; DLS, dynamic light scattering; EVs, extracellular vesicles; ER, endoplasmic reticulum; EPCs, endplate chondrocytes; FC, flow cytometry; iMSCs, induced pluripotent stem cell-derived MSCs; MATN3, matrilin-3; NP-MSCs, nucleus pulposus-derived mesenchymal stem cells; NP-MSCs, nucleus pulposus-derived mesenchymal stem cells; NPCs, nucleus pulposus cells; NA, not available; NTA, nanoparticle tracking analysis; SEM: scanning electron microscopy; TEM, transmission electron microscopy; IVDD, intervertebral disc degeneration; USCs, urine-derived stem cells; UPR, unfolded protein response; PLMSCs, placenta-derived mesenchymal stromal cells; UC-MSCs, umbilical cord-derived mesenchymal stem cells; WB, western blot.

### Matrix Synthesis and Degradation

ECM provides mechanical and chemical support to cells, contributing to the structural framework and homeostasis of connective tissues. The current evidence has indicated that intercellular interactions could be disrupted physically and functionally *via* EV-mediated proteolytic activities. EVs have been reported to contain matrix-remodeling enzymes including matrix metalloproteinases (MMPs), heparanases, hyaluronidases, and extracellular matrix metalloproteinase inducer, as well as aggrecanases such as adamalysin metalloproteinases having disintegrin and thrombospondin domains and tissue inhibitors of metalloproteinases (TIMPs), among others (Nawaz et al., 2018). It has been found that after simulation of SC-EVs, extracellular gene expression in degenerate NPCs significantly increases, such as Aggrecan, Collagen I or II, and SRY-Box transcription factor 9 (SOX9) (Lu et al., 2017; Zhu et al., 2020b). Also, SC-EVs downregulate the expression of MMPs, whereas upregulating TIMP-1, suggesting a more balanced anabolism/catabolism. In addition to regulating the proliferation activity of NPCs negatively, acidic pH disturbs the metabolic balance of ECM (Ohshima and Urban, 1992). In the pathological acid environment, SC-EVs promoted ECM synthesis and reduced ECM degradation by downregulating related enzymes (Li et al., 2020b). For instance, EVs derived from BM-MSCs or umbilical cord MSCs or were found to be able to protect NPCs or nucleus pulposus-derived MSCs (NP-MSCs) from high glucose-induced ECM degradation *via* the p38 MAPK pathway (Qi et al., 2019; Cui and Zhang, 2021).

EV therapy is a promising therapeutic approach for IVDD. However, the rapid clearance and disruption of EVs are the two primary challenges for the application of EV therapy in IVDD. Recently, a thermosensitive acellular ECM hydrogel coupled with adipose-derived MSCs (ASC) EVs were found to be able to regulate matrix synthesis and degradation, mitigating the inflammatory response, and ameliorating IVDD both *in vitro* and *in vivo* (Xing et al., 2021). Moreover, in a 3D pellet culture of DCs, after stimulation of SC-EVs, the researchers observed ECM production as early as day 7 and more than three times higher than the control group (Hingert et al., 2020). In addition, SC-EVs inhibited the secretion of MMP-1 in DCs. Interestingly, EVs derived from NPCs could recruit BM-MSCs and induce them to differentiate toward NP-like cells (Lu et al., 2017). In addition, there may be other interactions between NPCs and other cells inside the IVD such as AF or CEPs. These findings give an insight into new tissue regeneration treatment strategies to be developed for IVDD. Further studies should be focused on uncovering the role of EVs in matrix synthesis and degradation.

### Oxidative Stress and Inflammation

Previous studies have shown that oxidative stress (OS) and inflammation are extensively presented in IVDD (Feng et al., 20172017; Chao-Yang et al., 2021). For instance, inflammation was shown to increase in a rat IVDD model, which could be mitigated by SC-EVs *via* maintaining microenvironment hemostasis (Xing et al., 2021). Advanced glycation end products (AGEs) usually accumulate in aging and degenerative tissues, especially in collagen-rich ones, and are closely associated with endoplasmic reticulum (ER) stress and apoptosis (Liao et al., 2019). It was found that SC-EVs could reduce NPC AGE-induced ER stress and inhibit excessive activation of the unfolded protein response (UPR), subsequently alleviating apoptosis (Liao et al., 2019). The main pathways involved are reported to be AKT and ERK pathways. In another study, a pneumatic pressure model was constructed to mimic pressure-associated IVD tissue damage (Xiang et al., 2020). In this study, urine-derived SCs were utilized to separate EVs, inhibiting NPC ER stress-induced cell apoptosis in a dose-dependent manner. Of note, the study did not conduct any sorting analysis to remove urine-derived SCs from general urine-derived cells, which may affect the results (Ji et al., 20172017). Similarly, a study on lyophilized MSC-EVs showed that the EVs could counteract the OS damage induced by H_2_O_2_ on NPCs at concentrations between 5 and 50 mg/ml but become cytotoxic to NPCs at a concentration of over 50 mg/ml (Bari et al., 2018).

Mitochondria is an important organelle for cellular homeostasis, playing a crucial role in aging and disease development (Kang et al., 2020). Also, it is the predominant place to generate reactive oxygen species (ROS), which are also a source of organelle injury that can bring about cell damage as a secondary consequence of OS. It was reported that SC-EVs could not only suppress ROS production and NLRP3 inflammasome activation but also replenish mitochondrial-related proteins and attenuate mitochondrial dysfunction in NPCs (Xia et al., 2019; Zhang et al., 2020b).

Autophagy is the main intracellular degradation system that transports cellular components into autophagosomes for degradation, recycling, and reuse to keep renovation and homeostasis (Levine and Kroemer, 2019). Under physiological conditions, autophagy is found to be at a low level in NPCs and AF cells which are not degenerative. This suggests that autophagy is involved in the progress of IVDD. When IL-1β was applied to induce inflammation and apoptosis of AF cells, SC-EVs significantly inhibited the expression of autophagy-related proteins (Beclin-1 and LC3-II/I) by activating PI3K/AKT/mTOR signaling pathway (Li et al., 2020a). The beneficial effects could be reversed by rapamycin, an activator of autophagy. The results confirm OS and inflammation as potential targets, and SC-EVs could be a promising therapeutic strategy.

### miRNAs and Preconditions

As the crucial mediators for the benefits of SC-EVs, miRNAs are known to impact the expression of 30% of protein-coding genes mainly at the post-transcriptional level and numerous intracellular processes (Cazzanelli and Wuertz-Kozak, 2020). miRNAs can produce a sustained therapeutic effect and fundamental changes of the local microenvironment. In a study investigating EVs derived from umbilical cord MSCs, at least 100 kinds of miRNAs were found in EVs, among which the top 10 miRNAs with the highest abundance were miR-221–3p/222–3p, miR-574–3p, let-7a-5p, miR-23a-3p, miR-146a-5p, miR-320a, miR-193b-3p, miR-125a-5p, and miR-21–5p (Qi et al., 2019). However, the authors did not explore the functions of certain miRNAs further. Recently, many studies have investigated the role of miRNAs in SC-EVs for IVDD (Cheng et al., 2018; Zhu et al., 2020a; Zhu et al., 2020b; Xie et al., 2020; Yuan et al., 2020; Cui and Zhang, 2021; Sun et al., 2021; Wen et al., 2021). Anti-apoptotic miRNAs in SC-EVs include miR-105–5p, miR-129–5p, miR-199a, miR-21, miR-31–5p, miR-4450 inhibitor, miR-532–5p, and miR-142–3p for IVDD.

As mentioned above, some pathways mediate the beneficial effects of SC-EVs, while miRNAs regulate the expression of target genes in these pathways. For instance, miR-21 in SC-EVs suppressed NPC apoptosis by targeting phosphatase and tensin homolog (PTEN) through the PI3K-AKT pathway (Cheng et al., 2018). SC-EVs miR-142–3p alleviated NPCs injury by suppressing MAPK signaling by targeting MLK3 (Zhu et al., 2020a). Induced pluripotent stem cell-derived EVs exerted anti-aging effects by delivering miR-105–5p to senescent NPCs and activating the Sirt6 pathway (Sun et al., 2021). BM-MSC-EVs promoted proliferation and inhibited apoptosis and senescence *via* miR-199a targeting Gremlin 1 (GREM1) to downregulate the TGF-*β*pathway (Wen et al., 2021). Of note, through autocrine EVs, TGF-β was upregulated in CESCs, indicating different roles of TGF-β in different cells.

The inflammatory factors, such as tumor necrosis factor-alpha (TNF-α), played an important role in the pathological processes of IVDD. It has been reported that miR-532–5p is significantly downregulated in IVDD patients (Zhu et al., 2020b). BM-MSC-derived EVs transfer miR-532–5p to NPCs, suppressing apoptosis, ECM degradation, and fibrosis deposition *via* targeting RASSF5 (Zhu et al., 2020b). Macrophages play crucial roles in physiological conditions and inflammatory responses (Cunha et al., 2018). Polarization of M1 macrophages can precipitate local inflammation and structural change in IVDD. Interestingly, delivery of miR-129–5p to NPCs by BM-MSC-derived EVs led to a decline in apoptosis, ECM degradation, and M1 polarization of macrophages *via* targeting LRG1 and suppressing the p38 MAPK signaling pathway (Cui and Zhang, 2021).

Compared to the BM-MSCs, placenta-derived MSCs (PLMSCs) were shown to be more unlimited differentiation potential, therapeutic effects, and lower immunogenicity (Ni et al., 2014; Yuan et al., 2020). Therefore, PLMSC-derived EVs were used as nanocarriers to deliver miR-4450 inhibitors to increase NPC proliferation and migration while decreasing apoptosis by upregulating zinc finger protein 121 (ZNF121) (Yuan et al., 2020). Consistent with *in vitro* studies, *in vivo* experiments displayed the inhibitory effect of miR-4450 inhibitor in PLMSC-derived EVs, retarding IVDD damage.

As the nutrition channel, CEPs play a significant role as a barrier to nutrient transport in the endplate. In IVDD, CEPs degeneration results in malnutrition, leading to a degeneration of the NPCs and failed endogenous repair subsequently. To explore potential therapeutic methods, miR-31–5p in SC-EVs was found to be able to inhibit apoptosis and calcification under OS by targeting ATF6 in endplate chondrocytes (Xie et al., 2020). Similarly, EVs secreted by NPCs could promote NP-MSCs chondrogenic differentiation by delivery of miR-15a, downregulating MMP-3 through PI3K/AKT, and Wnt3a/β-catenin axis (Zhang et al., 2021d).

As a form of programmed cell death, pyroptosis, triggered by various inflammasomes such as NLRP3 or NLRP1, is also found in IVDD (Zhao et al., 2021). Recently, miR-410 in SC-EVs was proven to be a regulator of NLRP3. After delivery of miR-410 in SC-EVs, pyroptosis was significantly inhibited in IVDD (Zhang et al., 2020b). Similarly, miR-26a-5p in SC-EVs was reported to be another regulator of NLRP3, which targets METTL3, a methyltransferase mediating N6-methyladenosine (m6A), the most common endogenous RNA modification in mammalian cells (Yuan et al., 2021).

Recipient cells accept exogenous EVs usually *via* an endocytosis route (Mulcahy et al., 2014). However, the mechanism for EVs engulfed by NPCs is unknown. Recently, a study found that the EV uptake in NPCs is mediated by caveolae-dependent endocytosis, and caveolae-associated protein 2 (Cavin-2) plays a critical role in the process (Liao et al., 2021a). Also, Cavin-2-engineered EVs could not only protect against TNF-α-induced NPCs pyroptosis *in vitro* but also retard the progression of IVDD in the *ex vivo* disc culture model.

**FIGURE 3:**
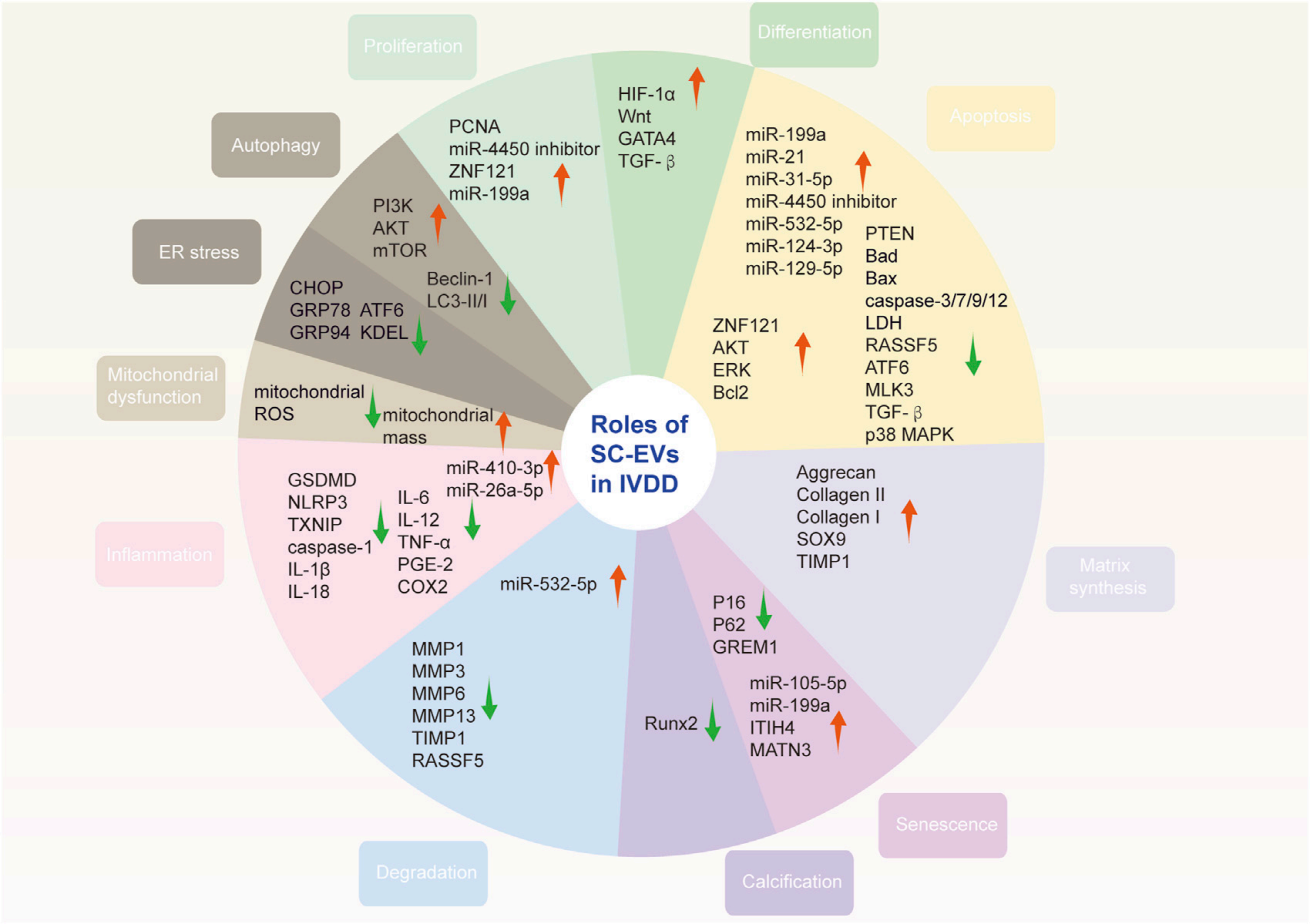
Roles of stem cell-derived extracellular vesicles in intervertebral disc degeneration. SC-EVs ameliorate IVDD *via* suppressing apoptosis and senescence as well as promoting proliferation. They also promote ECM synthesis and inhibit degradation. In addition, oxidative stress and inflammation are reduced after SC-EVs delivered. Note: red arrow, upregulated; green arrow, downregulated; SC-EVs, stem cell-derived extracellular vesicles; IVDD, intervertebral disc degeneration; ECM, extracellular matrix.

Metformin is the most commonly prescribed drug for type 2 diabetes (Flory and Lipska, 2019). Besides, it is an AMPK activator and is closed to aging diseases and autophagy (Wang et al., 2018). Recently, a study found that metformin could not only promote MSC-derived EVs secretion but also yield better quality (Liao et al., 2021b). The positive was found to be associated with the autophagy-related pathway. Furthermore, metformin induced the phosphorylation of synaptosome-associated protein 29 (SNAP29), a critical soluble N-ethylmaleimide sensitive fusion factor attachment protein receptor (SNARE) protein that mediates the fusion of multivesicular bodies with the plasma membrane, facilitating the release of EVs. Through functional analysis, EVs derived from metformin-treated BM-MSCs significantly ameliorated NPCs senescence (Figure 3).

## Conclusions and Future Perspectives

The present review reveals overall positive findings and may result in an increasing interest in the current emerging field. Although SC-EVs represent a more accessible and cell-free therapy, some challenges should be taken into consideration before translation to clinical applications such as production, safety, long-term durability, and regulation (Bari et al., 2018; Xing et al., 2021). First of all, many different procedures to separate EVs have been described in the literature. Unfortunately, a standardized separation process is still lacking, leading to uncertainty about EVs biological effects and safety, which is strongly influenced by the preparation methods (Théry et al., 2018). Future studies should focus on improving separation and enrichment techniques, formulating standardized procedures for the large-scale production of SC-EVs, ready-to-use products, suitable to replace SCs in regenerative nanomedicine. Also, it is of importance to differentiate the different subtypes of EVs in heterogeneous samples qualitatively and quantitatively such as chromatography techniques, tangential flow, and microfluidics. A series of procedures and regulations need to be formulated and carried out based on MISEV 2018.

Secondly, the rapid clearance and disruption of EVs are two major challenges when applying SC-EVs in IVDD. Also, the mode of administration is also of importance for the application of SC-EVs including systematic or local injections. One-shot may not ensure the alleviation of the degeneration process for a long time, whereas repeated injections may destroy the structure of the intervertebral disc. Recently, a thermosensitive acellular ECM hydrogel was reported to serve as a powerful platform for IVDD drug or EV delivery (Xing et al., 2021). Future studies could focus on different materials combined with EVs to be a biotherapy and an alternative therapy for IVDD. Preconditions and modified EVs, especially genetically engineered EVs, could be another method to target specific genes to treat IVDD safely and efficiently.

Thirdly, although EVs have been administered to humans already in the early 2000s to treat cancer patients, there are still no standard techniques established for the clinical-grade production and quality control of EV-based therapeutics so far. Hence, another critical need will be to develop assays that predict the therapeutic potency of SC-EVs in quantifiable, robust, and reproducible parameters for clinical applications. Previous studies have emphasized quantifiable assays to define the identity of SC-EVs and requirements for prospective potency assays to predict the effectiveness and safety (Lener et al., 2015; Witwer et al., 2019; Gimona et al., 2021). Although all the included studies rest on the preclinical stage, current evidence for cell-free therapy reveals the feasibility of revolutionizing traditional therapeutic alternatives for IVDD in the future.
